# Assembling of Fluid Filtration System for Quantitative Evaluation of Microleakage in Dental Materials

**Published:** 2008-07-10

**Authors:** Maryam Javidi, Neda Naghavi, Ehsan Roohani

**Affiliations:** 1 *Department of Endodontics, Dental School, Dental Research Center, Mashad University of Medical Sciences, Mashad, Iran*; 2 *Department of Endodontics, Dental School, Mashad University of Medical Sciences, Mashad, Iran*; 3 *Department of Restorative Dentistry, Dental School, Mashad University of Medical Sciences, Mashad, Iran*

**Keywords:** Bacterial Leakage, Dye Leakage, Fluid Filtration, New Device

## Abstract

**INTRODUCTION:** There are several methods for evaluating microleakage in dentistry, for example dye or bacterial leakage, electro-chemical methods, radioisotope labeling and fluid filtration. The purpose of this study was to assemble the fluid filtration system for quantitative evaluation of microleakage in dental materials.

**MATERIALS AND METHODS:** The roots were connected to a tube filled with an underwater pressure supply. A bubble was introduced into the water to measure endodontic leakage. A digital camera and professional software were utilized to record and measure the bubble displacement.

**RESULTS:** Our system was constructed successfully and functioned correctly.

**CONCLUSION:** In this pilot study we found this system efficient for the evaluation of microleakage of dental materials.

## INTRODUCTION

The fluid filtration method, introduced and developed by Pashley *et al. *has been extensively used for 20 years for understanding the physiology of dentin (1), as well as the affects of various restorative treatments on dentin permeability (2). Microleakage, whether apical or coronal, is still a clinical problem and a possible cause of failure in endodontic treatments. Leakage studies have become very popular in dental research and many methods and devices have been introduced for microleakage evaluation. The introduction of the fluid filtration method in endodontics (3), has gained popularity in evaluating apical or coronal microleakage. Many researchers have evaluated the sealing efficiency of root end filling materials (4-8), apical leakage of post and core restorations in endodontically treated teeth (9) and the microleakage of temporary restorative materials (10). This method has also been used to compare the sealing efficiency of different root canal filling methods (11-13).

This method presents several advantages over the common dye penetration method: the samples are not destroyed, permitting the evaluation of sealing efficiency over time; the results are automatically records avoiding any operator bias; the results are very accurate because very small volumes can be recorded (14) and no tracer is needed with the related problems of molecular size, affinity for dentin, or pH as major problems in the dye penetration method. No intricate materials are required as in bacterial penetration studies or radioactive tracer studies (15).

However, there is no standardization of the materials and methods. The pressure used ranged from 10 to 20 psi, 0.1 to 5 atm or 15 to 30 cm H_2_O, and the measurement time ranges from 1 min to 3 h (16). The result is generally expressed as µl/min/cm H_2_O.

The purpose of this study was to assemble the fluid filtration system for quantitative evaluation of microleakage in dental materials.

**Figure 1:**
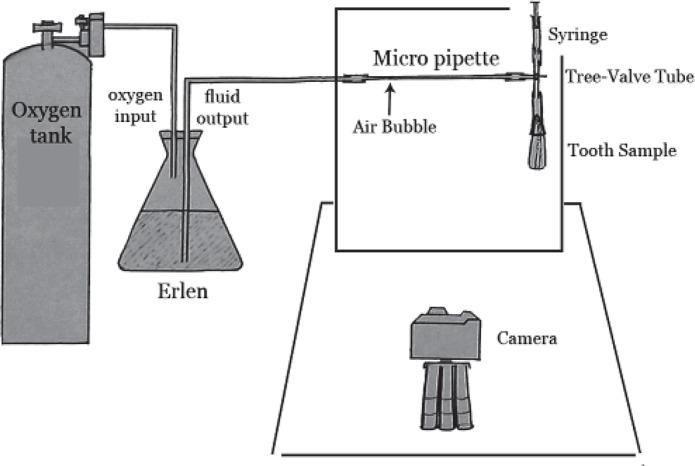
A schematic view of new designed system

## MATERIALS AND METHODS

This system involves the evaluation of fluid transport in specimens calculated from bubble movement. It is necessary to apply pressure to fluid to move through the specimen and displace the bubbles. Therefore an oxygen tank equipped with a manometer (to precise adjustment of pressure) was utilized. A specific plastic tube was connected to the oxygen source and the end part was connected to an Erlen. Two holes were made on the Erlen's cap, one for the entrance of oxygen and the other for emersion of fluid (Figure 1). The oxygen tube was fixed above the fluid in the Erlen, but the glass cylinder was entirely immersed in water (the end that removes the fluid) and the other side of this cylinder was connected to a micropipette (0.1 cc) by a plastic tube. This micropipette was fixed on a vertical plate and its other end was connected to a three-valve tube by a latex pipe (0.5 cm in diameter and approximately 2 cm in length). The three-valve tube was equipped with a bilateral control faucet; when turned on, only two directions were connected.

The upper side of the three-valve tube was connected to a syringe, which was used to create an air bubble through the micropipette. The diameter of the air bubble should not be smaller than the internal diameter of the micropipette so its movement will be a precise indicator for fluid movement in the micropipette.

The lower side was used to connect specimens (discussed below). All of the connections of this system were smeared with cyanoacrylate glue (Inter Lock Co., Japan) and covered by multiple layers of Parafilm strips (Parafilm M, Laboratory Film, Chicago, USA). This strip seals connections in experimental tubes to guarantee an impervious connection.

Micropipette, connections and the three-valve tube were fixed on the vertical plate of the system made of compressed plastic. This vertical plate was mounted on a completely stable horizontal MDF plate (22×34 cm in diameters). The role of this horizontal plate was to provide stability for the vertical plate and to communicate between two parts of the system; the two parts are as outlined below (Figure 1).

Part 1 consists of tubes, micropipette, pipes and a tooth sample that transfer pressure to the specimen and Part 2 contains a recorder of fluid transport. For the first time we used a digital camera (Olympus, C765, 4 MP, Japan) and a professional software (AutoCAD 2006, Autodesk, Inc.) in this system to record and measure the amount of bubble displacement. We mounted the camera on the horizontal plate using a small tripod with a distance of 32 cm from the vertical plate. It was placed on the middle of the micropipette to prevent minimal errors. Its distance from the micropipette was the closest possible to allow the camera to cover the entire range of the bubble's movement.

The outer surfaces of all teeth except the apical 2 mm should be covered with Parafilm strips. We considered two negative control samples to ensure that there was no leakage or fluid movement within the device. These teeth samples were intact with the outer side of the root, crown and the apical foramen entirely covered by multiple layers of Parafilm strips.

In the positive control samples the root was not covered (the canals were instrumented but not obturated), this is to ensure that all system paths were opened without obstruction.

The apical end of the root excluding apical foramen was covered by cyanoacrylate glue and inserted in a latex pipe (Guihua Co., China) with a 0.5 cm internal diameter and 5 cm length. The free end of the pipe was connected to the only free end of the three-valve tube (the lower end).

This junction was sealed completely by Parafilm strip. The control faucet was closed toward the tooth, so only the syringe and the micropipette was connected. We used the syringe to insert an appropriate air bubble to the micropipette.

Once this was carried out the sample was ready for experiment. Its relevant number was written near the micropipette. The camera was adjusted in the macrograph to take precise picture in a short distance. The control faucet was opened to the tooth and the syringe was removed from the pass. Now only the tooth and the fluid filtration system were connected. Then the major faucet of the oxygen tank was opened. The pressure was previously adjusted, since it should be constant during all steps of the experiments.

The first picture of the bubble position in the micropipette was taken after 30 sec to reach a balance in the system. Four subsequent pictures were taken at 2-minute intervals (2, 4, 6 and 8 min after the first one). At the end of the experiments, the faucet for the oxygen tank and then the control faucet to the samples were closed. The Parafilm strips were opened and the latex pipe was disconnected from the three-valve tube. The same steps were repeated for the next samples.

Five pictures of each sample were transferred to the computer. The bubble position in each picture was determined by professional software (Auto CAD 2006). The precision of this software was 0.1 mm, so with the device used in this study, the smallest recordable displacement was 0.031 mm and the smallest recordable volume was about 10^-8^ liter.

Displacement measures were introduced to custom-made software designed for accelerating the calculations. This software calculates the mean displacement of the bubble per minute and then with a specific quotient converts the longitudinal displacement of the bubble into the volume of fluid passing from the samples showing it as µl/min/cm H_2_O unit. Thus one number for each sample represented the amount of leakage in the canal (µl/min/cm H_2_O).

## RESULTS

There was no bubble displacement in the negative control samples, but in the positive control samples as soon as the major faucet of the oxygen tank was opened fluid flow was observed. In this pilot study the method utilized was efficient for the evaluation of microleakage in dental materials.

## DISCUSSION

The purpose of this study was to assemble a novel fluid filtration system. There is an absence of a standard fluid filtration system. Wimonchit *et al. *used a pressure of 100 mmHg for evaluation of different methods for leakage assessment (15), Pommel *et al. *used a pressure of 15 cm H_2_O (17), Bobotis *et al. *used a pressure of 20 psi for the evaluation of leakage of temporary filling materials (18), and in the study of Abramovitz, the applied pressure was 1.2 atm (19). Researchers have used different methods to produce the needed pressure for their study. We used O_2_ pressure because it remains unchanged during the experiment and is adjustable for different clinical situations.

Also we used a digital camera for recording the bubble displacement because of its extreme precision and as it permits the rereading the fluid displacement. It is also time saving.

This method is based on forcing liquid through the sealer or its existing voids, dentin walls and gutta-percha. Therefore the results may follow the laws governing the phenomena of filtration, such as the Poiseuille-Hagen equation, and may be modified by factors like applied pressure or measurement time (1). The compliance of the system and the initial filling of microscopic voids may explain the decrease in fluid flow over time. Compliance is the change in volume of a system for every unit change in pressure. For instance a large bubble may compress more than a small one, and long elastic tubing may expand more than small rigid ones. These changes would be exaggerated at high pressures. The compliance of the system may be of concern for a small measurement of time but not if one waits for enough time for the fluid under pressure in the system to reach a steady state with regards to bubble compression and tubing expansion (1).

Several parameters modify the outcome of the work: the diameter of tube containing the bubble, time and applied pressure. In fact, the time factor was not chosen for its clinical relevance but rather for the convenience of the researcher. This does not pose a problem as the purpose of this study was to draw comparisons between different techniques or different materials; not to correlate the results to a clinical situation. Thus the amount of time should be as long as possible to increase the accuracy of the measurement without leading to exaggerated and useless results.

We conducted this pilot study to:

1- Have confidence in non-leaking connections;

2- Make calibration of the system and allow primary adjustment such as oxygen pressure;

3- Evaluate the efficacy of camera used for recording the result;

4- Evaluate the ability of software in reading the results;

5- Predict probable problems that may occur.

As mentioned before, our software calculates the mean displacement of a bubble per minute and then using a specific quotient converts the longitudinal displacement of the bubble into the volume of fluid current from the samples and shows it as µl/min/cm H_2_O. This quotient is the sum of the quotients outlined below.

1- Camera's magnification ratio: a digital camera with a resolution of 5 megapixels has a constant magnification when photos are taken in an invariable distance. We calculated this magnification and used it as a quotient.

2- Length to volume conversion ratio: 1 mm displacement of bubble in the pipette is equivalent to a constant volume of fluid movement in the sample. This ratio was applied as a quotient too.

3- The atmosphere to cm H2O conversion ratio: we decided to represent the results as µl/min/ cm H_2_O to accelerate comparing them with results of other studies.

It is noteworthy that we can change the applied pressure, the time of experiment or the number of intervals according to the protocol of this study; the only changes being the data entered into the software. Since the recorded volume is divided by the measurement time and the pressure to express the results (ie, µl/min/the unit of pressure), there is a linear relationship between the volume, time measured and pressure.

## CONCLUSION

This system was assembled successfully for quantitative evaluation of microleakage in dental materials, with the precision of 10^-^^1^Lit for the smallest recordable volume and as a result, we had one number for each sample that represented the amount of leakage as µl/min/cm H_2_O.
